# Role of pain and anxiety in mediating relationships between donation history and vasovagal reaction symptoms in blood donors in England

**DOI:** 10.1111/trf.70004

**Published:** 2025-11-21

**Authors:** Yaning Wu, Emanuele Di Angelantonio, Stephen Kaptoge, Angela M. Wood, Philippe T. Gilchrist, Matthew Walker, Nathalie Kingston, Barbara Masser, David Roberts, Eamonn Ferguson, Lois G. Kim

**Affiliations:** ^1^ British Heart Foundation Cardiovascular Epidemiology Unit, Department of Public Health and Primary Care University of Cambridge Cambridge UK; ^2^ Victor Phillip Dahdaleh Heart and Lung Research Institute University of Cambridge Cambridge UK; ^3^ National Institute for Health and Care Research Blood and Transplant Research Unit in Donor Health and Behaviour University of Cambridge Cambridge UK; ^4^ Health Data Science Research Center Human Technopole Milan Italy; ^5^ School of Psychological Sciences Macquarie University Sydney New South Wales Australia; ^6^ Lifespan Health and Wellbeing Research Center Macquarie University Sydney New South Wales Australia; ^7^ Department of Haematology University of Cambridge Cambridge UK; ^8^ National Institute for Health and Care Research BioResource for Translational Research Cambridge UK; ^9^ Research & Development Australia Red Cross Lifeblood West Melbourne Australia; ^10^ School of Psychology The University of Queensland Brisbane Queensland Australia; ^11^ National Health Service Blood and Transplant John Radcliffe Hospital Oxford UK; ^12^ Radcliffe Department of Medicine University of Oxford Oxford UK; ^13^ School of Psychology University of Nottingham Nottingham UK; ^14^ Pain Center Versus Arthritis University of Nottingham Nottingham UK

**Keywords:** anxiety, blood donation, mediation analysis, pain, vasovagal reactions

## Abstract

**Background:**

Vasovagal reactions (VVRs; faintness or fainting) can harm donor health and retention. Higher VVR rates are often observed in first‐time donors and donors with VVR histories. We quantified associations between donation history (including donation experience, donation frequency, and VVR history) and VVR symptom reports in donors in England and assessed their mediation by venipuncture pain and donation anxiety.

**Methods:**

In 60,026 STRIDES BioResource study participants recruited from 2019 to 2022, donation history was obtained from blood service records, while venipuncture pain, donation anxiety, and VVR symptoms were reported via post‐donation questionnaires. We conducted causal mediation analyses estimating risk ratios (RRs) for indirect effects of donation history on VVR symptom reports through pain and anxiety while quantifying exposure–mediator interaction.

**Results:**

Adjusted RRs for VVR symptoms were 1.24 (95% confidence interval: 1.19, 1.30) for newer/lapsed donors, 1.19 (1.13, 1.25) for less frequent donors, and 1.82 (1.71, 1.94) for donors with VVR histories. Pain and anxiety were associated with up to 1.28 and 1.60 times the risk of symptom reporting. Anxiety mediated 19.0% and 11.2% of associations with donation experience and frequency, whereas pain mediated no associations. Associations of pain and anxiety with VVR symptoms were only observed among donors without, not with, VVR histories.

**Discussion:**

Our findings suggest that differences in venipuncture pain and donation anxiety do not primarily explain differences in VVR symptoms by blood donation history. While intervening on pain and anxiety may fail to equalize symptom disparities linked to donation history, interventions may reduce VVR burden in donors without VVR histories.

**Abbreviations**
AMT: applied muscle tension
BDAS: Blood Donor Anxiety Scale
BDRI: blood donation reactions inventory
CI: confidence interval
IQR: interquartile range
MICE: multiple imputation by chained equations
NDE: natural direct effect
NHS: National Health Service
NHSBT: National Health Service Blood and Transplant
NIE: natural indirect effect
NIHR: National Institute for Health and Care Research
OR: odds ratio
PM: proportion mediated
RERI: relative excess risk due to interaction
RR: risk ratio
SF‐36: 36‐Item Short Form Survey
STRIDES: Strategies to Improve Donor Experiences
TE: total effect
VVR: vasovagal reaction

## 1 INTRODUCTION

While whole blood donation is generally safe, vasovagal reactions (VVRs), that is, feelings of faintness with or without loss of consciousness, occur in 0.1%–7.0% of donations.^1^ VVRs may result in injury, deter donor return, and incur costs and medicolegal liabilities for blood services.^1^ Therefore, identifying at‐risk donors and developing preventive interventions is paramount to blood supply sufficiency.

Published studies have observed higher VVR risk in first‐time donors and those with a history of donation‐related VVRs.^2^, ^3^, ^4^, ^5^ Additionally, donation‐related pain,^6^, ^7^ anxiety,^6^, ^7^, ^8^, ^9^, ^10^ and fear^11^, ^12^, ^13^, ^14^, ^15^, ^16^, ^17^ have been associated with higher VVR risk. Neurobiological mechanisms such as habituation (i.e., decreasing responses to stimuli over multiple harmless exposures)^18^ and aversive conditioning (intensifying responses to stimuli following a harmful exposure) may change these negative responses over multiple donations.

We hypothesize that differences in VVR risk across donation history characteristics (i.e., donation experience, donation frequency, and VVR history) operate, at least partially, through habituation and aversive conditioning of pain and anxiety. Though published studies have associated donation history with pain, anxiety, and VVRs, no study has attempted to disentangle the complex relationships between these constructs, new insights into which may inform preventive interventions on a blood‐service‐wide scale. Such studies require statistical examination of associations and mechanisms that leverage large‐scale data with validated measurements.

We analyzed data on up to 60,026 whole blood donors in England to quantify associations of donation experience, donation frequency, and VVR history with subsequent VVR symptom reports. We aimed to assess potential mediation of these associations by venipuncture pain and donation anxiety while quantifying exposure‐mediator interactions.

## 2 METHODS

### 2.1 Study population

We analyzed data from the Strategies to Improve Donor Experiences (STRIDES) BioResource, a sample of voluntary whole blood donors attending 73 National Health Service Blood and Transplant (NHSBT) donation sites across England between March 25th, 2019 and November 3rd, 2022. Eligibility criteria for both donation and STRIDES BioResource participation included good general health, age 17 years or older, and weight between 50 and 158 kg.

Participants completed online questionnaires collecting demographic, medical history, and donation‐related characteristics at least 1 week after the donation during which they were enrolled in the study (henceforth, their “index donation”). Written informed consent from participants was obtained at enrollment and prior to questionnaire completion.^19^

### 2.2 Exposures

We defined three binary donation history exposures using routine blood service records. First, we defined a donation experience indicator comparing donors who had been donating for less than 2 years or had not donated in the 2 years prior to study enrollment (“newer/lapsed donors”) with donors who had been donating for 2 or more years and had donated at least once in the 2 years prior to study enrollment (“experienced donors”). A 2‐year cutoff was chosen to capture donor lapse in accordance with blood service definitions (with lapsed donors, defined by NHSBT as individuals giving no donations in the previous 2 years, treated similarly to new donors on session^20^, ^21^) in addition to donor career length. Second, we defined a donation frequency indicator comparing donors giving less than versus more than or equal to sex‐specific median donations (four donations for male and three donations for female donors) during the previous 2 years among donors who donated at least once during those 2 years (i.e., “experienced donors” in donation experience analyses), using a median cutoff to maximize these analyses’ statistical power. Third, we constructed a VVR history indicator comparing donors with and without any history of phlebotomist‐recorded VVRs (henceforth, “previously reacting” and “VVR‐naïve” donors, respectively) during the 5 years preceding study enrollment. We used this cutoff to maximize statistical power given that only 0.6% of the analytic sample experienced more than one VVR during the 5 years prior to study enrollment.

### 2.3 Outcome

Participants recalled VVR symptoms at their index donation via the Blood Donation Reaction Inventory (BDRI),^5^ a validated four‐item questionnaire assessing faintness, dizziness, weakness, and lightheadedness using six‐point Likert scales from “not at all” to “to an extreme degree”. This measure has demonstrated concurrent validity with phlebotomist‐assessed VVRs.^5^ Given the lack of a validated scale cutoff, BDRI scores were dichotomized to compare donors reporting no and any VVR symptoms in this analysis to capture a wide spectrum of symptom severities.

### 2.4 Mediators

Venipuncture pain at the index donation was assessed using a numeric rating scale from 0 to 100 in response to the question “How painful was the donation needle going into your arm?”. Responses were dichotomized into “mild‐or‐less pain” (<30) and “worse‐than‐mild pain” (≥30) in accordance with a published threshold used in clinical settings.^22^

Donation anxiety was measured using the validated six‐item Blood Donor Anxiety Scale (BDAS),^23^ which contained six items relating to present or absent anxiety using three‐point Likert scales from “not at all” to “very much.” To align with published evidence suggesting that absence‐based scale items may not capture underlying constructs,^24^ only three scale items indicating present anxiety (“tense,” “nervous,” and “jittery”) were used. Total scores were then dichotomized to compare donors reporting no and any anxiety given the absence of a validated scale cutoff, capturing a range of anxiety severities.

### 2.5 Covariates

Potential confounders of exposure, mediator, and outcome relationships were identified from the published literature. They included sex (male/female),^25^ age (years),^25^ ethnicity (White/non‐White),^25^ estimated blood volume (EBV, liters, calculated from donor‐reported height and weight using Nadler's equation^26^),^25^ general‐practitioner‐diagnosed hypotension (yes/no),^27^ smoking (never/former/current smoker),^28^ alcohol intake (never/former/occasional/regular drinker),^28^ leisure time physical activity (inactive/moderately inactive/moderately active/very active),^27^ general health (0–100, derived from the 36‐item Short Form Survey [SF‐36] questionnaire^29^),^30^ blood draw fear (any/none, dichotomized from a five‐point Likert scale),^6^ and recent nervousness (five‐point Likert scale, derived from the SF‐36).^31^ Confounders specific to donation frequency and VVR history analyses included the number of donations in the 3 years preceding exposure assessment and 5 years preceding study enrollment, respectively.

### 2.6 Statistical analyses

Analyses of donation experience included all participants with available donation history data. Analyses of donation frequency were restricted to participants who had made at least one donation in the 2 years preceding study enrollment to ensure comparability of exposure groups. To minimize reverse causality, previously reacting donors were excluded from donation experience and frequency analyses. Additionally, first‐time donors at baseline were excluded from analyses of VVR history.

**FIGURE 1:**
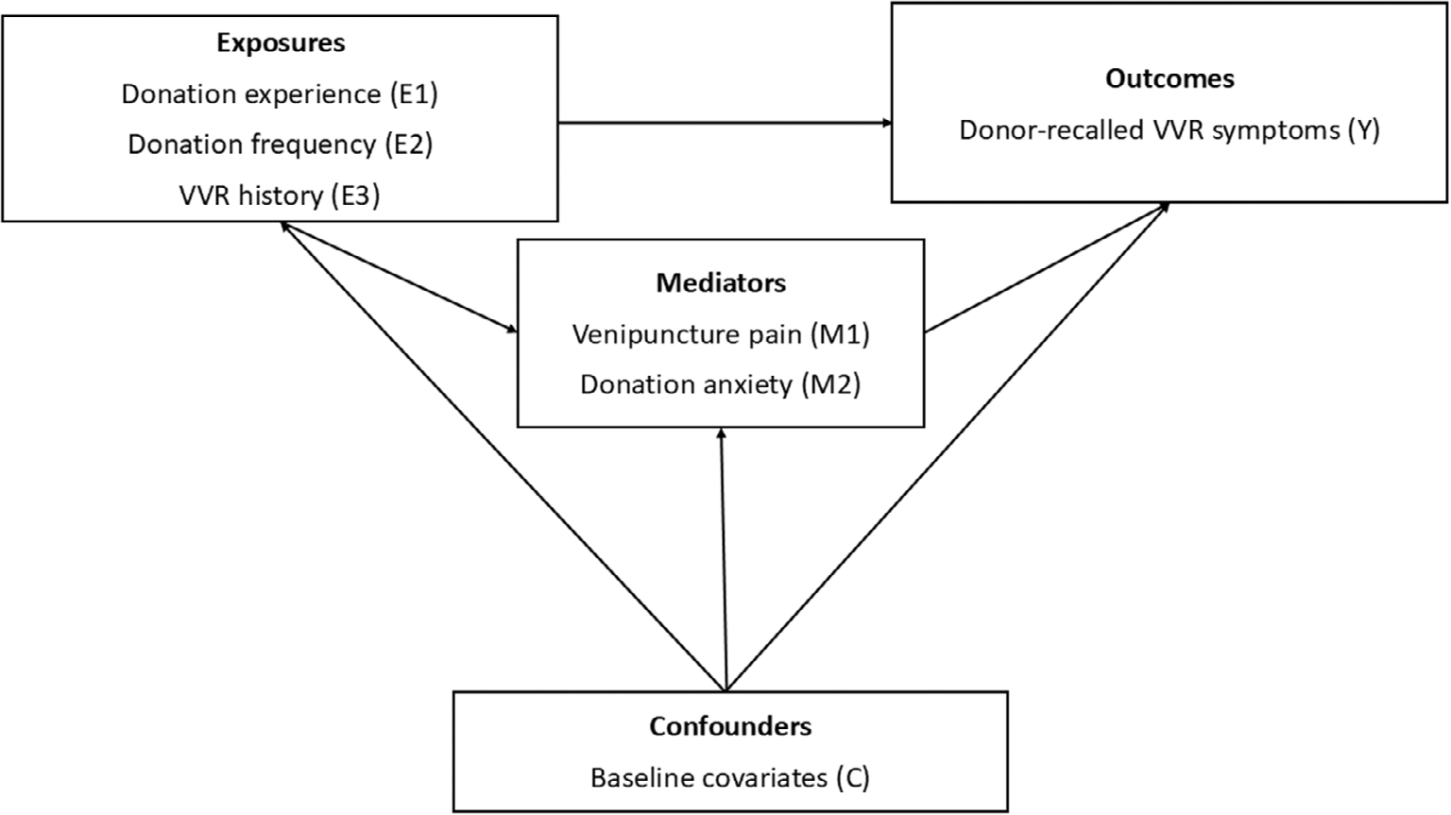
Causal direct acyclic graphs for analyses of donation experience, donation frequency, and vasovagal reaction history. Donation experience (E1) analyses compared individuals who had been donating for <2 years or had not donated in the 2 years prior to study enrollment (“newer/lapsed donors”) and individuals who had been donating for ≥2 years and had donated at least once in the 2 years prior to study enrollment (“experienced donors”). Donation frequency (E2) analyses compared individuals giving ≤ versus > sex‐specific median donations (four donations for male and three donations for female donors) in the 2 years prior to study enrollment. VVR history (E3) analyses compare individuals experiencing at least one versus zero phlebotomist‐recorded vasovagal reaction (VVRs) in the 5 years prior to study enrollment.

Hypothesized relationships between all three donation history characteristics and VVR symptoms are displayed in directed acyclic graphs in Figure 1. Multivariable log‐binomial regression models were used to estimate relative risks (RRs) and 95% confidence intervals (CIs) for associations between donation history exposures and VVRs. Multivariable logistic regression models were used to estimate odds ratios (ORs) and 95% CIs for associations between these exposures and pain and anxiety. Causal mediation analyses evaluated the extent to which associations between donation history exposures and VVR symptom reports were explained by pain and anxiety, respectively.^32^ Unlike traditional difference and product methods,^33^ causal mediation methods can accurately estimate direct and indirect effects in the presence of exposure‐mediator interaction. Identified estimates assume no unmeasured exposure‐outcome, mediator‐outcome, and exposure‐mediator confounding and no mediator‐outcome confounders influenced by the exposure.^32^

For each donation history exposure, we estimated total effects (TE), natural direct effects (NDE, representing associations independent of pain or anxiety), natural indirect effects (NIE, representing mediation through pain or anxiety), and proportions mediated (PM, representing the proportion of the TE operating through a mediator and calculated using ${\mathrm{RR}}^{\mathrm{NDE}}\left({\mathrm{RR}}^{\mathrm{NIE}}-1\right)/\left({\mathrm{RR}}^{\mathrm{NDE}}\times {\mathrm{RR}}^{\mathrm{NIE}}-1\right)$).^32^ Standard errors for TEs, NDEs, NIEs, and PMs were calculated using the delta method, assuming a normal distribution of each estimator in the presence of a sufficiently large sample (previously defined as *N* > 1000^34^). Across analyses of all three donation history exposures, we additionally estimated effect modification of relationships between pain and anxiety and VVR symptoms and quantified exposure‐mediator interaction on the additive (through relative excess risks due to interaction [RERI]^35^) and multiplicative scales.

For all analyses, we multiply imputed missing data using models including all analytic variables (Appendix S1). We also conducted additional and sensitivity analyses to evaluate our findings' robustness to varying mediator severities, a fully validated donation anxiety scale, and a complete‐case approach to data missingness (Appendix S2). All analyses were conducted using R v4.4 (R Core Team, 2024).

## 3 RESULTS

### 3.1 Participant characteristics

**FIGURE 2:**
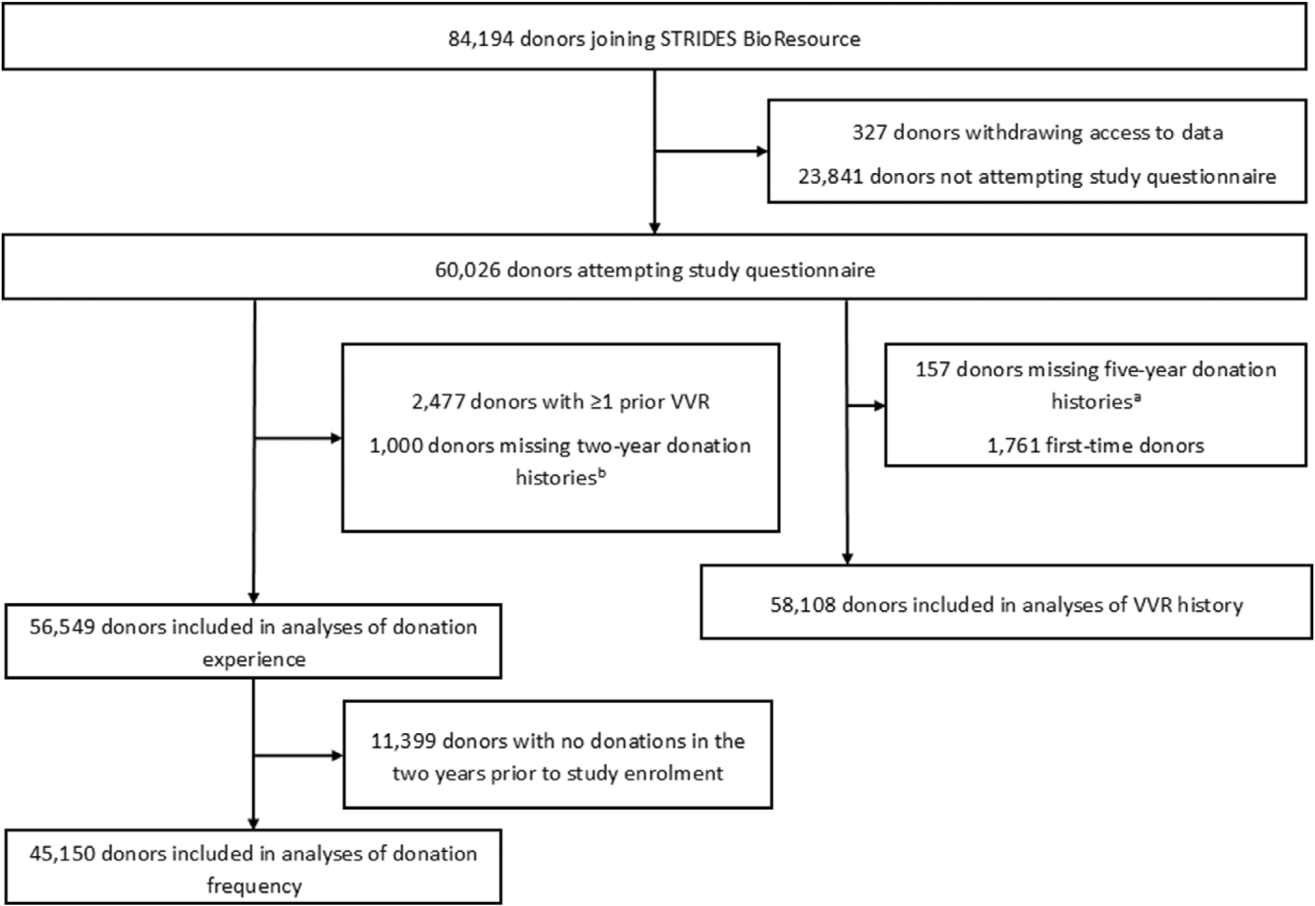
Selection of participants of the STRIDES BioResource study (2019–2022) into the analytic sample. ^a^Data on number of donations and vasovagal reaction (VVRs) in the 5 years prior to study enrollment. ^b^Data on number of donations and VVRs in the 2 years prior to study enrollment. Missingness indicates that data were not available on whether donations given during 5 years prior to study enrollment were given during the 2 years immediately prior to enrollment.

Of the 84,194 donors originally recruited into the STRIDES BioResource, 327 donors (0.4%) withdrew permission to use their data for research. Of the remaining 83,867 participants, 60,026 (71.5%) completed the baseline questionnaire. Figure 2 displays the participant inclusion flow for analyses of all three donation history exposures.

Newer/lapsed and less frequent donors were more likely to be younger and from non‐White ethnic backgrounds (*p* < .001; Tables 1 and 2). Previously reacting donors were generally younger, more likely to be female, and had smaller EBVs (*p* < .001; Table 3).

**TABLE 1:** Baseline characteristics of analytic sample for analyses of donation experience (*N* = 56,549).

Characteristic	*N*	Experienced donors^a^	*N*	Newer/lapsed donors^a^	*p*‐value^b^
				*N* = 11,399^a^	
Donor‐recalled VVR symptoms at index donation	44,470	6540 (15%)	11,249	2894 (26%)	<.001
Worse‐than‐mild venipuncture pain	44,466	5665 (13%)	11,112	1710 (15%)	<.001
Any donation anxiety	43,580	7728 (18%)	11,103	3796 (34%)	<.001
Age (years)	45,150	50 (37, 59)	11,399	38 (28, 50)	<.001
Female sex	45,150	22,635 (50%)	11,399	5602 (49%)	.059
White ethnicity	44,761	42,231 (94%)	11,386	9884 (87%)	<.001
Estimated blood volume (liters)	43,006	4.89 (4.18, 5.52)	10,823	4.92 (4.19, 5.55)	.015
Diagnosed hypotension	43,945	3435 (7.8%)	10,817	882 (8.2%)	.2
Smoking status	43,749		10,725		<.001
Never		28,392 (65%)		6591 (61%)	
Former		13,497 (31%)		3407 (32%)	
Current		1860 (4.3%)		727 (6.8%)	
Alcohol intake	42,004		10,433		<.001
Never		1044 (2.5%)		363 (3.5%)	
Former		3751 (8.9%)		1054 (10%)	
Occasional		10,333 (25%)		3032 (29%)	
Regular		26,876 (64%)		5984 (57%)	
Leisure time physical activity	44,689		11,220		<.001
Inactive		555 (1.2%)		180 (1.6%)	
Moderately inactive		5477 (12%)		1513 (13%)	
Moderately active		27,159 (61%)		6588 (59%)	
Very active		11,498 (26%)		2939 (26%)	
Any blood draw fear	44,891	5348 (12%)	11,287	2305 (20%)	<.001
General health score	43,337	75 (65, 85)	10,743	75 (65, 85)	<.001
“Nervousness” in past 4 weeks	44,251		10,960		<.001
All of the time		144 (0.3%)		62 (0.6%)	
Most of the time		859 (1.9%)		443 (4.0%)	
Some of the time		5798 (13%)		2053 (19%)	
A little of the time		12,509 (28%)		3663 (33%)	
None of the time		24,941 (56%)		4739 (43%)	

Abbreviation: VVR, vasovagal reaction.

^a^ “Experienced donors” defined as donors who had been donating for at least 2 years prior to study enrollment and who donated at least once in the 2 years prior to study enrollment. “Newer/lapsed donors” defined as donors who had been donating for less than 2 years or had not donated in the 2 years prior to study enrollment. Descriptive statistics given as median (interquartile range [IQR]), *N* (%).

^b^ Pearson's chi‐squared test; Wilcoxon rank sum test.

**TABLE 2:** Baseline characteristics of analytic sample for analyses of donation frequency (*N* = 45,150).

Characteristic	*N*	More frequent donors^a^	*N*	Less frequent donors^a^	*p*‐Value^b^
Donor‐recalled VVR symptoms at index donation	29,661	3757 (13%)	15,079	2783 (18%)	<.001
Worse‐than‐mild venipuncture pain	29,468	3598 (12%)	14,998	2067 (14%)	<.001
Any donation anxiety	28,846	4368 (15%)	14,734	3360 (23%)	<.001
Age (years)	29,922	51 (39, 60)	15,228	47 (34, 58)	<.001
Female sex	29,922	15,131 (51%)	15,228	7504 (49%)	.01
White ethnicity	29,629	28,249 (95%)	15,132	13,982 (92%)	<.001
Estimated blood volume (liters)	28,497	4.89 (4.18, 5.52)	14,509	4.90 (4.18, 5.50)	.9
Diagnosed hypotension	27,738	2184 (7.5%)	14,733	1251 (8.5%)	<.001
Smoking status	29,086		14,663		<.001
Never		19,071 (66%)		9321 (64%)	
Former		8867 (30%)		4630 (32%)	
Current		1148 (3.9%)		712 (4.9%)	
Alcohol intake	27,885		14,119		.001
Never		669 (2.4%)		375 (2.7%)	
Former		2443 (8.8%)		1308 (9.3%)	
Occasional		6749 (24%)		3584 (25%)	
Regular		18,024 (65%)		8852 (63%)	
Leisure time physical activity	29,622		15,067		<.001
Inactive		348 (1.2%)		207 (1.4%)	
Moderately inactive		3476 (12%)		2001 (13%)	
Moderately active		18,077 (61%)		9082 (60%)	
Very active		7721 (26%)		3777 (25%)	
Any blood draw fear	29,760	3199 (11%)	15,131	2149 (14%)	<.001
General health score	28,792	75 (65, 85)	14,545	75 (65, 85)	<.001
“Nervousness” in past 4 weeks	29,389		14,862		<.001
All of the time		86 (0.3%)		58 (0.4%)	
Most of the time		492 (1.7%)		367 (2.5%)	
Some of the time		3560 (12%)		2238 (15%)	
A little of the time		8036 (27%)		4473 (30%)	
None of the time		17,215 (59%)		7726 (52%)	
Number of donations in the 3 years prior to exposure assessment	29,922	6 (4, 8)	15,228	4 (2, 6)	<.001

Abbreviation: VVR, vasovagal reaction.

^a^ “More frequent donors” is defined as donors who had donated more than or equal to sex‐specific medians (four donations for male donors and three donations for female donors) within the analytic sample in the 2 years prior to study enrollment. “Less frequent donors” is defined as donors who had donated less than sex‐specific medians in the 2 years prior to study enrollment. Descriptive statistics are given as median (IQR), *N* (%).

^b^ Pearson's chi‐squared test; Wilcoxon rank sum test.

**TABLE 3:** Baseline characteristics of analytic sample for vasovagal reaction history (*N* = 58,108).

Characteristic	*N*	VVR‐naïve donors^a^	*N*	Previously reacting donors^a^	*p*‐Value^b^
Donor‐recalled VVR symptoms at index donation	55,107	9098 (17%)	2433	1128 (46%)	<.001
Worse‐than‐mild venipuncture pain	54,713	7244 (13%)	2421	501 (21%)	<.001
Any donation anxiety	53,812	10,934 (20%)	2404	1132 (47%)	<.001
Age (years)	55,644	48 (35, 58)	2464	35 (26, 48)	<.001
Female sex	55,644	27,831 (50%)	2464	1527 (62%)	<.001
White ethnicity	55,241	51,510 (93%)	2458	2277 (93%)	.2
Estimated blood volume (liters)	52,974	4.90 (4.18, 5.52)	2348	4.54 (3.98, 5.27)	<.001
Diagnosed hypotension	53,942	4241 (7.9%)	2456	261 (11%)	<.001
Smoking status	53,670		2369		<.001
Never		34,488 (64%)		1613 (68%)	
Former		16,659 (31%)		647 (27%)	
Current		2523 (4.7%)		109 (4.6%)	
Alcohol intake	51,651		2320		<.001
Never		1352 (2.6%)		79 (3.4%)	
Former		4726 (9.1%)		232 (10%)	
Occasional		13,106 (25%)		715 (31%)	
Regular		32,467 (63%)		1294 (56%)	
Leisure time physical activity	55,039		2434		<.001
Inactive		735 (1.3%)		25 (1.0%)	
Moderately inactive		6872 (12%)		368 (15%)	
Moderately active		33,266 (60%)		1475 (61%)	
Very active		14,166 (26%)		566 (23%)	
Any blood draw fear	55,296	7335 (13%)	2448	803 (33%)	<.001
General health score	53,263	75 (65, 85)	2354	75 (65, 85)	<.001
“Nervousness” in past 4 weeks	54,379		2407		<.001
All of the time		200 (0.4%)		10 (0.4%)	
Most of the time		1260 (2.3%)		118 (4.9%)	
Some of the time		7661 (14%)		510 (21%)	
A little of the time		15,905 (29%)		812 (34%)	
None of the time		29,353 (54%)		957 (40%)	
Number of donations in the 5 years prior to study enrollment	55,644	8 (4, 11)	2464	6 (3, 10)	<.001

Abbreviation: VVR, vasovagal reaction.

^a^ “VVR‐naïve donors” defined as donors who did not experience a phlebotomist‐recorded VVR in the 5 years prior to study enrollment. “Previously reacting donors” defined as donors who experienced at least one phlebotomist‐recorded VVR in the 5 years prior to study enrollment. Descriptive statistics given as median (IQR), *N* (%).

^b^ Pearson's chi‐squared test; Wilcoxon rank sum test.

### 3.2 Associations between donation history, pain and anxiety, and VVRs

**FIGURE 3:**
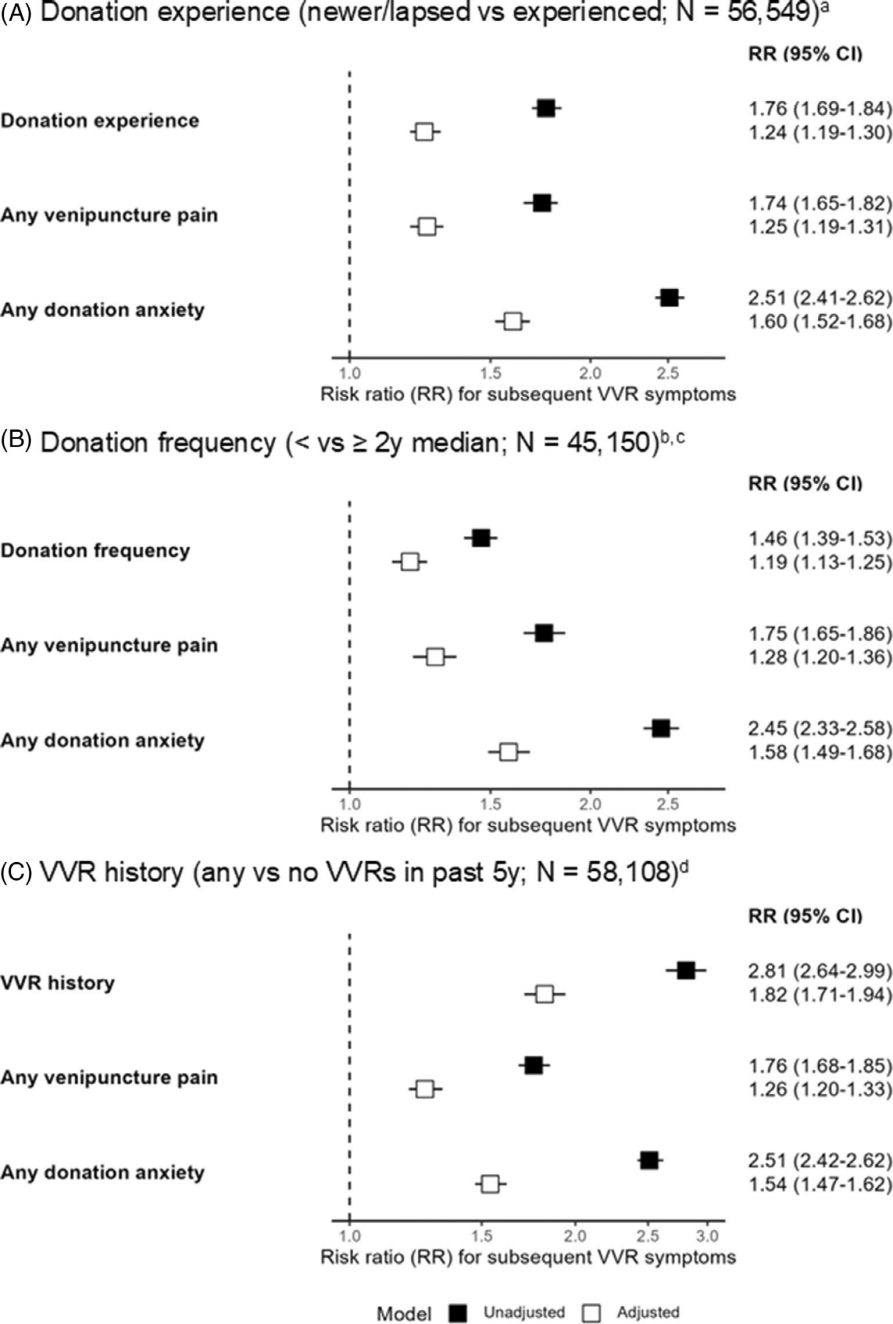
Associations between donation history exposures, venipuncture pain and donation anxiety, and vasovagal reaction symptoms. CI, confidence interval; RR, risk ratio; VVR, vasovagal reaction. ^a^Adjusted for sex, age, ethnicity, estimated blood volume, diagnosed hypotension, smoking, alcohol intake, leisure time physical activity, general health, blood draw fear, and recent nervousness. ^b^Sex‐specific 2‐year donation frequency medians were four donations in male donors and three donations in female donors respectively. ^c^Adjusted for sex, age, ethnicity, estimated blood volume, diagnosed hypotension, smoking, alcohol intake, leisure time physical activity, general health, blood draw fear, recent nervousness, and number of donations in the 3 years before exposure assessment period. ^d^Adjusted for sex, age, ethnicity, estimated blood volume, diagnosed hypotension, smoking, alcohol intake, leisure time physical activity, general health, blood draw fear, recent nervousness, and number of donations in the 5 years prior to study enrollment.

We observed higher risks of VVR symptom reporting among newer/lapsed versus experienced donors (RR: 1.24; 95% CI: 1.19, 1.30) and less versus more frequent donors (1.19; 1.13, 1.25), as well as previously reacting versus VVR‐naïve donors (1.82; 1.71, 1.94) (Figure 3). Furthermore, donors who reported worse‐than‐mild venipuncture and any donation anxiety had up to 1.28 (1.20, 1.36) and 1.60 (1.52, 1.68) times higher risk of reporting any VVR symptoms, respectively, compared with donors reporting mild‐or‐less pain and no donation anxiety (Figure 3). Although newer/lapsed, less frequent, and previously reacting donors had higher odds of reporting donation‐related anxiety, only previously reacting donors had higher odds of reporting worse‐than‐mild venipuncture pain compared to VVR‐naïve donors (Table S2).

### 3.3 Mediation and interaction analyses: venipuncture pain

Mediation analyses for venipuncture pain were only conducted for VVR history as this was the only donation history exposure positively associated with venipuncture pain in multivariable regression analyses.^36^ There was no evidence of mediation by venipuncture pain (NIE RR: 1.00; 95% CI: 1.00, 1.00; Table 4), suggesting that pain does not explain the observed difference in VVR symptom reporting risk between previously reacting and VVR‐naïve donors.

**TABLE 4:** Mediation and interaction analyses for donation experience, donation frequency, and vasovagal reaction history.

Mediator: venipuncture pain						
	Adjusted^a^ RRs (95% CIs)			% mediated	Additive interaction	Multiplicative interaction
Exposure	TE	NDE	NIE			
Donation experience^b^	–	–	–	–	−0.08 (−0.21, 0.05) *p* = .22	0.90 (0.81, 1.00) *p* = .06
Donation frequency^c^	–	–	–	–	0.11 (−0.01, 0.22) *p* = .02	1.05 (0.93, 1.18) *p* = .44
VVR history	1.87 (1.75, 2.00)	1.86 (1.75, 1.99)	1.00 (1.00, 1.00)	0.0 (−0.4, 0.4)	−0.30 (−0.46, −0.14) *p* < .001	0.77 (0.67, 0.89) *p* < .001

Mediator: donation anxiety						
	Adjusted^a^ RRs (95% CIs)			% mediated	Additive interaction	Multiplicative interaction
Exposure	TE	NDE	NIE			
Donation experience	1.27 (1.21, 1.33)	1.20 (1.15, 1.26)	1.04 (1.03, 1.05)	19.0 (12.7, 25.2)	−0.05 (−0.17, 0.07) *p* = .45	0.90 (0.82, 0.98) *p* = .02
Donation frequency	1.19 (1.13, 1.26)	1.17 (1.11, 1.23)	1.02 (1.01, 1.02)	11.2 (6.4, 16.1)	0.02 (−0.08, 0.12) *p* = .75	0.95 (0.86, 1.06) *p* = .34
VVR history	2.02 (1.88, 2.18)	1.90 (1.77, 2.04)	1.01 (0.99, 1.02)	1.6 (−1.6, 4.9)	−0.41 (−0.61, −0.21) *p* < .001	0.69 (0.61, 0.78) *p* < .001

Abbreviations: CI, confidence interval; NDE, natural direct effect, or the association between each donation history exposure and VVR symptom reports operating independently of pain or anxiety; NIE, natural indirect effect, or the association between each donation history exposure and VVR symptom reports operating via pain or anxiety; RR, risk ratio; TE, total effect, or the association between each donation history exposure and VVR symptom reports; VVR, vasovagal reaction.

^a^ All analyses adjusted for sex, age, ethnicity, estimated blood volume, diagnosed hypotension, smoking, alcohol intake, leisure time physical activity, general health, blood draw fear, and recent nervousness. Analyses of donation frequency and VVR history additionally adjusted for numbers of donations in the 3 years prior to the exposure assessment period and the previous 5 years prior to study enrollment, respectively.

^b^ Mediation analyses were not conducted because donation experience showed a weak inverse association with venipuncture pain following covariate adjustment (see Table S2).

^c^ Mediation analyses not conducted because donation frequency showed a null association with venipuncture pain following covariate adjustment (see Table S2).

Analyses of effect modification of relationships between pain and VVR symptoms found a moderate positive association among VVR‐naïve donors (RR: 1.30; 95% CI: 1.24, 1.37; Table S3) but no evidence for an association among previously reacting donors (1.00; 0.87, 1.14; Table S3). Strong evidence for negative interaction on both additive and multiplicative scales was found (*p* values for interaction <.001; Table 4).

### 3.4 Mediation and interaction analyses: donation anxiety

Mediation analyses identified donation anxiety as a partial mediator between both donation experience and frequency and VVR symptom reporting. Specifically, anxiety mediated 19.0% (12.7, 25.2) and 11.2% (6.4, 16.1) of associations of donation experience and frequency with VVR symptom reports, respectively. Conversely, anxiety did not mediate differences in VVR symptom reporting risk between previously reacting and VVR‐naïve donors (NIE RR: 1.01; 95% CI: 0.99, 1.02; Table 4).

Analyses of effect modification of relationships between anxiety and VVR symptoms found a moderate positive association among VVR‐naïve donors (RR: 1.55; 95% CI: 1.48, 1.63; Table S3) and little evidence for an association among previously reacting donors (1.06; 0.94, 1.20; Table S3). Strong evidence for negative interaction on both additive and multiplicative scales was found (*p* values for interaction <.001; Table 4).

### 3.5 Additional and sensitivity analyses

Analyses categorizing anxiety by severity yielded NIEs consistent with primary analyses across all three donation history exposures (Table S4). In addition, analyses using a dichotomized version of the full six‐item BDAS and taking a complete‐case approach to missing data produced broadly similar results to primary analyses (Tables S5 and S6).

## 4 DISCUSSION

Our study observed higher frequencies of vasovagal reaction symptom reports among newer/lapsed, less frequent, and previously reacting blood donors in England. Venipuncture pain and donation anxiety explained less than one‐fifth of these associations. Furthermore, the relationships of pain and anxiety with VVR symptom reporting appeared to vary by donors' VVR history status, with moderate positive associations seen only in VVR‐naïve donors. Although interventions addressing donation‐related pain and anxiety may have little impact on VVR symptom disparities by donation history, such interventions could reduce overall symptom burden in the VVR‐naïve donor population.

These observations broadly align with previously published research that has reported higher VVR rates among first‐time donors^3^, ^4^ and previously reacting donors,^2^, ^3^ as well as positive associations of blood draw fear,^11^, ^12^, ^13^, ^14^, ^15^, ^17^ donation anxiety,^6^, ^7^, ^8^, ^9^, ^10^ and venipuncture pain^6^, ^7^ with VVRs. Consistent with prior literature observing greater fear in less experienced donors,^6^, ^37^ we observed elevated anxiety among newer/lapsed donors compared to experienced donors, though we observed similar levels of venipuncture pain across donors regardless of donation history contrary to one US‐based published study.^38^ Moreover, though the INTERVAL trial found no difference in VVRs across donors in England who were randomized to differing donation frequencies,^39^ trial participants were a highly self‐selected donor sample who may have been less vulnerable to VVRs at baseline. Notably, our analyses extend existing knowledge by examining differences in VVR symptom reports by individuals' time‐bounded, rather than lifetime, donation frequency. Our examination of recent donation frequency, analyzed independently of cumulative donations given, may support the collection of time‐bounded donation data when appraising individual donors' risks of reporting VVR symptoms.

Interpretations of associations of greater donation experience and frequency with lower risks of reporting VVR symptoms and donation anxiety should be cautious due to possible self‐selection biases, analogous to the “healthy worker survival effect” observed in occupational epidemiology.^40^ Given the voluntary nature of blood donation in England, individuals prone to VVRs may selectively cease donation early, potentially leading to reverse causation between donation experience and frequency and individuals' vulnerability to VVRs and/or anxiety. We attempted to mitigate biases associated with VVR vulnerability by excluding donors with VVR histories from analyses of donation experience and frequency—published path analyses have shown that associations of fear and anxiety with donor return operate almost exclusively via VVR occurrence at an index donation.^7^, ^11^, ^41^ Furthermore, our novel analyses of donation frequency as a donation history exposure should have minimized the influence of biases arising from selective donor drop‐out on associations with both VVR symptom reports and donation anxiety by comparing donors who donated with differing intensities during similar follow‐up periods. Moreover, small studies observing decreasing cortisol responses following multiple donations^42^ and non‐donation venipunctures^42^ support the presence of a “true” habituation effect in donation anxiety across donor careers. Therefore, donor self‐selection is unlikely to entirely explain observed associations of donation experience and frequency with subsequent VVR symptoms and anxiety. Nonetheless, the inability of our dataset to detect previous donor drop‐outs following a VVR may have masked the occurrence of sensitization, or increased negative responses to donation stimuli following an initial adverse event, in these donors had they returned. This phenomenon may be especially prevalent in less experienced donors.^43^

Our findings of null associations of both pain and anxiety with VVR symptom reporting in previously reacting donors may, too, be attributable to selection biases. In analyses of VVR history, the present study's analytic sample excluded donors who did not return after experiencing a phlebotomist‐recorded VVR. Published qualitative data from previously reacting donors in Australia suggest that donors who identified a physiological cause of their reaction (e.g., poor pre‐donation hydration or nutrition) were more likely to return than donors who attributed their reaction to donation‐related anxiety.^44^ Previously reacting donors included in the present analyses, given their returning status, may have therefore been more vulnerable to VVRs driven by physiological, rather than psychological, etiologies. This may have produced null associations of pain and anxiety with VVR symptom reports in this group. However, these null associations may also be attributable to differential appraisal of VVR symptom questionnaires by previously reacting and VVR‐naïve donors. Previously reacting donors may have been more likely to report VVR symptoms at their index study donation if they attributed their symptoms to a vasovagal response (as opposed to characterizing them as anxiety, which often shows similar symptomology^45^). Therefore, responses such as pain and anxiety may have been relatively decoupled from VVR symptom reporting in this subgroup. Broadly, large‐scale data on return determinants, risk factor profiles, and symptom perceptions in previously reacting donors are needed to fully understand the mechanisms underlying reported VVR recurrence.

Our findings suggest that interventions targeting donation‐related pain and anxiety might help reduce VVR symptoms in donors without VVR histories. However, published evidence for the efficacy of psychologically targeted VVR interventions is conflicted. One small trial using a tablet‐based psychosocial leaflet showed no effect on phlebotomist‐reported VVRs.^6^, ^46^ Conversely, interventions involving direct staff support may hold more promise, though evidence is sparse.^47^ Evidence for the effectiveness of direct interventions on venipuncture pain is also negligible, though some blood services currently provide local anesthesia at the venipuncture site.^48^ Finally, multiple randomized trials have shown the effectiveness of applied muscle tension (AMT) exercises, which are thought to tackle both psychological and physiological etiologies of VVRs.^49^ Further work remains necessary to identify effective and scalable psychosocial interventions for VVRs.

Our analyses suggest that donation history does not primarily influence VVR risk via venipuncture pain or donation anxiety—other mechanisms may therefore also be salient. For example, higher rates of VVR symptom reporting in donors with VVR histories may be explained by increased attention toward similar bodily sensations at subsequent donations.^50^ In addition, lower rates of symptom reporting in experienced and more frequent donors may be explained by greater engagement with VVR‐preventive interventions, such as hydration and AMT,^49^ in this group. Conversely, a qualitative study found that previously reacting donors may be more willing to engage in preventive strategies in future donations,^44^ implying that behavioral mechanisms may be less relevant to VVR symptom disparities by VVR history status. Furthermore, donation history disparities in symptom reports may also be explained by changes in pre/peri‐donation physiological parameters such as heart rate and/or blood pressure given the etiologic proximity of these cardiovascular markers to VVR occurrence.^27^

Our study has several strengths, including its large sample size, diversity in donor characteristics, inclusion of both static and mobile donation sites, validated self‐reported measures, and comprehensive statistical adjustments for confounders. Furthermore, analyses employed a unified causal framework to disentangle links between donation history and subsequent VVR symptoms while allowing for exposure‐mediator interactions. Finally, additional and sensitivity analyses supported the broad robustness of findings to varying mediator severities, measurement methods, and approaches to missing data handling.

Several potential limitations should also be acknowledged. First, the cross‐sectional nature of these data precludes definite causal interpretations. In particular, the simultaneous measurement of pain, anxiety, and VVR symptoms may have artificially inflated concordance between these measures given donors' pre‐formed schema (i.e., knowledge frameworks) about the interrelatedness of these constructs.^51^ However, these inflations are unlikely to have been substantial given the relatively modest associations observed in this study of both mediators with VVR symptom reports. Nevertheless, pre‐/peri‐donation measurements of pain and anxiety are necessary to clarify temporal relationships. Second, the severity of donor‐reported VVR symptoms could have been falsely inflated in donors with long questionnaire response lag times.^52^, ^53^ However, recall bias was unlikely to have substantially influenced analytic findings given that the present study dichotomized the BDRI and that 92% of BioResource participants completed the study questionnaire within 4 weeks of their index donation. Nevertheless, using phlebotomist‐recorded VVRs in future analyses may improve upon the present study's self‐reported measure. Third, we treated blood draw fear as a baseline confounder in mediation models despite potential evolution of fear across donation careers, meaning that this variable may have been a mediator‐outcome confounder affected by the exposure. Adjustment for this variable may have blocked direct paths between donation history variables and VVR symptom reports, leading to overestimated indirect effects for both pain and anxiety.^32^ However, this broadly supports our conclusion that pain and anxiety do not explain the majority of donation history disparities in VVR symptoms. Fourth, in fitting separate mediation models for venipuncture pain and donation anxiety, we assumed that these mediators were independent conditional on covariates.^32^ However, anxiety may heighten donors' appraisal of venipuncture pain.^6^ Analyses of mediation of VVR history associations by pain may have therefore violated the aforementioned assumption relating to exposure‐induced mediator‐outcome confounding.^32^ However, true indirect effects are likely small given the magnitude of associations of pain with VVR history and VVR symptom reports. Notably, summing indirect effects of pain and anxiety would likely overestimate the proportion mediated by these factors due to shared causal paths.^32^ Fifth, unmeasured exposure‐outcome, mediator‐outcome, and exposure‐mediator confounding may have persisted due to this study's observational design. Finally, this study's analytic sample was not entirely representative of all donors in England; study participants were comparatively older, were more likely to be female and White, and gave more previous donations at baseline.^54^

In conclusion, this nationwide study of whole blood donors in England identified associations of newer/lapsed donor status, lower donation frequency, and previous VVR occurrence with higher rates of subsequent VVR symptom reports. Venipuncture pain did not explain these associations, whereas donation anxiety partially accounted for associations with donation experience and frequency. These findings highlight the need for increased vigilance for VVR symptoms among newer/lapsed, less frequent, and previously reacting donors. Moreover, though minimizing pain and anxiety may not fully address differences in VVR symptoms associated with donation history, such strategies have the potential to broadly reduce symptoms among blood donors. Further randomized studies are therefore needed to identify appropriate interventions.

## FUNDING INFORMATION

Emanuele Di Angelantonio is supported by a NIHR Senior Investigator Award. Angela M. Wood is supported by the BHF Data Science Center (HDRUK2023.0239), Health Data Research UK (Big Data for Complex Disease‐HDR‐23012), and as an NIHR Research Professor (NIHR303137). Lois G. Kim is funded by the BHF Cambridge Center for Research Excellence RE/24/130011. This work was supported by core funding from the British Heart Foundation (RG/F/23/110103), NIHR Cambridge Biomedical Research Center (NIHR203312), BHF Chair Award (CH/12/2/29428), Cambridge BHF Center of Research Excellence (RE/18/1/34212) and by Health Data Research UK, which is funded by the UK Medical Research Council, Engineering and Physical Sciences Research Council, Economic and Social Research Council, Department of Health and Social Care (England), Chief Scientist Office of the Scottish Government Health and Social Care Directorates, Health and Social Care Research and Development Division (Welsh Government), Public Health Agency (Northern Ireland), British Heart Foundation and the Wellcome Trust.

## CONFLICT OF INTEREST STATEMENT

The authors have disclosed no conflicts of interest.

## Supporting information


**Data S1.** Supporting Information.

## Data Availability

Bona fide scientists can seek access to relevant de‐identified individual participant data from the STRIDES BioResource (and a copy of the study's data dictionary) by applying to the STRIDES Data Access Committee by contacting ceu‐dataaccess@medschl.cam.ac.uk. The STRIDES Data Access Committee reviews (supplemented, when required, by expertise from additional scientists external to the Committee) applications according to usual academic criteria of scientific validity and feasibility. Following approval by the Committee, a material transfer or research collaboration agreement will be agreed and signed with the applicants. Applicants will be required to provide updates to the STRIDES Data Access Committee on their use of STRIDES trial data, including provision of copies of any publications. Applicants will be required to adhere in publications to the STRIDES trial's policy for acknowledgment of the trial's funders, stakeholders, and scientific or technical contributors.
