# Supplementary material for: Role of pain and anxiety in mediating relationships between donation history and vasovagal reaction symptoms in blood donors in England
Source: Transfusion. 2025 Nov 21;66(1):131–45. doi: 10.1111/trf.70004 (PMC12857869; doi:10.1111/trf.70004)
Supplement: Supplementary file 1 — Data S1. Supporting Information. [file TRF-66-131-s001.docx]

**Supplementary material**

**Role of pain and anxiety in mediating relationships between donation history and vasovagal reaction symptoms in blood donors in England**

**Table of contents**

**Appendix S1.** Methods for multiple imputation of missing data.

**Appendix S2.** Rationale and conduct of additional and sensitivity analyses.

**Table S1.** Imputation methods used for analytic variables with missing values.

**Table S2.** Associations of donation history characteristics with venipuncture pain and donation anxiety.

**Table S3.** Risk ratios of venipuncture pain and donation anxiety with donor-reported VVR symptoms by donation experience, donation frequency, and VVR history subgroups.

**Table S4.** Mediation and interaction analyses using differing pain and anxiety severities.

**Table S5.** Mediation and interaction analyses for donation anxiety using six-item BDAS.

**Table S6**. Mediation and interaction analyses using a complete-case approach.

**Appendix S1**. Methods for multiple imputation of missing data.

~20% of data across analytic samples for all three donation history exposures were missing. Therefore, multiple imputation with chained equations (MICE) was implemented given its flexibility towards the imputation of multiple missing variables of various types and involves running multiple iterations of single-variable imputations.^1^ First, where $Y$ is a set of $k$ variables with partially observed values including ${(Y}_{1}, \ldots, Y_{k})$ and $Y_{i}$ denotes one of $k$ variables, $Y^{mis}$ (missing values of $Y$) were filled in by random draws from $Y^{obs}$ (observed values of $Y$)’s marginal distribution. For $Y_{1}$, for example, observed values were regressed on all other variables in the imputation model and missing values were imputed using draws from the variable’s corresponding posterior predictive distribution, which contained possible unobserved values of $Y_{1}$ conditional on all other variables. For ${(Y}_{2}, \ldots, Y_{k})$, an identical procedure was followed, with previously imputed variable values then treated as observed values within regressions. For all variables within $Y$, 10 iterations of this process were run to stabilise predicted values and generate a single imputed dataset. These iterations were then repeated $m$ times to generate $m$ complete datasets.^2,3^

Multiple imputations were conducted separately for each exposure-mediator pair (with a total of six sets of imputed datasets produced). Exposure, mediator, and outcome variables, as well as hypothesised exposure-outcome, mediator-outcome, and exposure-mediator confounders, were included in imputation models for respective mediation analyses to maximise the likelihood that important causes of missingness were taken into account to approximate a “missing at random” mechanism^4-6^ and avoid excessive loss of information. These analyses used logistic regression to impute binary variables, ordered logistic regression to impute ordered categorical variables, and predictive mean matching (using 10 candidate donor participants with complete data to minimise poor performance due to ties)^7,8^ to impute interval and ratio scale variables. **Table S1** describes the methods used to impute each analytic variable across analyses of donation experience, donation frequency, and VVR history. $m$ = 20 datasets were multiply imputed, with this value chosen to reduce uncertainty in imputed estimates by approximating the proportion of participants (~20%) with missing data for any analytic variable in analyses of all three donation history exposures. Regression and mediation analyses were subsequently conducted in each imputed dataset, with results pooled for each set of imputed datasets using Rubin’s rules.^2^

**Table S1**. Imputation methods used for analytic variables with missing values.

| **Imputation method** | **Variables** |
| --- | --- |
| Logistic regression | Donor-reported VVRs, venipuncture pain, donation anxiety, ethnicity, GP-diagnosed hypotension, blood draw fear |
| Ordered logistic regression | Smoking, alcohol intake, leisure time physical activity |
| Predictive mean matching | Estimated blood volume, general health, recent nervousness |

Note: Donation experience, donation frequency, donations in the past 3 years (used only in analyses of donation frequency), donations in the past 5 years (used only in analyses of VVR history), age, and sex contained no missing values.

**Appendix S2**. Rationale and conduct of additional and sensitivity analyses.

Additional analyses comparing multiple mediator severities

To assess the influence of mediator severity on mediation effects, additional analyses were conducted where venipuncture pain was further categorised into moderate (31/100-60/100) and severe (>60/100) pain based on validated numeric rating scale cutoffs,^9^ while donation anxiety was further categorised into moderate (1/9-5/9) and severe (>5/9) levels based on cut-offs for clinically relevant state anxiety originally validated in a shortened version of the STAI’s state anxiety subscale.^10^ Additional analyses then estimated the effect on VVR risk of a change in donation experience, donation frequency, and VVR history independent of and through a change in each mediator from the lowest exposure category (i.e., mild venipuncture pain or no donation anxiety) to each respective severity category conditional on covariates.

Sensitivity analyses using full version of donation anxiety measure

Given the original validation of the full, six-item version of the BDAS, this study’s donation anxiety measure, in voluntary donors rather than the three-item version dichotomised for use in this study’s main analyses,^11^ sensitivity analyses were conducted to assess the robustness of findings to the full version of the questionnaire. In these analyses, donors were classified as having any donation anxiety if they responded with Likert scale options other than “not at all” for negatively framed BDAS items and/or options other than “very much” for positively framed BDAS items.

Sensitivity analyses using a complete-case approach

To assess the robustness of analyses to missing data imputation using MICE, sensitivity analyses using a complete-case approach were conducted. In these analyses, methods identical to the main analyses were used to quantify mediation and interaction, with observations with missing data on exposures, mediators, outcomes, or covariates removed from the analytic sample.

**Table S2**. Associations of donation history characteristics with venipuncture pain and donation anxiety.

| **Exposure: Donation experience** (N = 56,549) | | | | | | | |
| --- | --- | --- | --- | --- | --- | --- | --- |
|  | Unadjusted model | | | Adjusted model^a^ | | | |
| Outcome | OR (95% CI) | *P* value | | OR (95% CI) | | *P* value | |
| Venipuncture pain | 1.25 (1.18, 1.32) | <.001 | | 0.92 (0.87, 0.98) | | .01 | |
| Donation anxiety | 2.42 (2.31, 2.54) | <.001 | | 1.84 (1.74, 1.94) | | <.001 | |
| **Exposure: Donation frequency** (N = 45,150) | | | | | | | |
|  | Unadjusted model | | | Adjusted model^a^ | | | |
| Outcome | OR (95% CI) | *P* value | | OR (95% CI) | | *P* value | |
| Venipuncture pain | 1.15 (1.08, 1.22) | <.001 | | 1.01 (0.94, 1.07) | | .85 | |
| Donation anxiety | 1.66 (1.58, 1.75) | <.001 | | 1.36 (1.28, 1.45) | | <.001 | |
| **Exposure: VVR history** (N = 58,108) | | | | | | |  |
|  | Unadjusted model |  | | Adjusted model^a^ | |  | |
| Outcome | OR (95% CI) | *P* value | OR (95% CI) | | *P* value | |  |
| Venipuncture pain | 1.71 (1.55, 1.89) | <.001 | 1.12 (1.01, 1.25) | | .03 | |  |
| Donation anxiety | 3.52 (3.24, 3.82) | <.001 | 2.14 (1.94, 2.37) | | <.001 | |  |

OR: odds ratio; CI: confidence interval; VVR: vasovagal reaction.

^a^All analyses adjusted for sex, age, ethnicity, estimated blood volume, diagnosed hypotension, smoking, alcohol intake, leisure time physical activity, general health, blood draw fear, and recent nervousness. Analyses of donation frequency and VVR history additionally adjusted for numbers of donations in the three years prior to the exposure assessment period and the previous five years prior to study enrolment respectively.

**Table S3**. Risk ratios of venipuncture pain and donation anxiety with donor-reported VVR symptoms by donation experience, donation frequency, and VVR history subgroups.

| **Analyses of donation experience** | | |
| --- | --- | --- |
|  | Adjusted^a^ RRs (95% CIs) | |
| Mediator | Newer and lapsed donors | Experienced donors |
| Venipuncture pain | 1.17 (1.07, 1.28) | 1.30 (1.22, 1.38) |
| Donation anxiety | 1.46 (1.35, 1.58) | 1.62 (1.53, 1.72) |
| **Analyses of donation frequency** | | |
|  | Adjusted^a^ RRs (95% CIs) | |
| Mediator | Less frequent donors | More frequent donors |
| Venipuncture pain | 1.31 (1.20, 1.44) | 1.25 (1.15, 1.36) |
| Donation anxiety | 1.53 (1.40, 1.66) | 1.60 (1.48, 1.73) |
| **Analyses of VVR history** | | |
|  | Adjusted^a^ RRs (95% CIs) | |
| Mediator | Donors with 1+ VVRs in the past 5y | Donors with no VVRs in the past 5y |
| Venipuncture pain | 1.00 (0.87, 1.14) | 1.30 (1.24, 1.37) |
| Donation anxiety | 1.06 (0.94, 1.20) | 1.55 (1.48, 1.63) |

RR: risk ratio; CI: confidence interval; VVR: vasovagal reaction.

^a^All analyses adjusted for sex, age, ethnicity, estimated blood volume, diagnosed hypotension, smoking, alcohol intake, leisure time physical activity, general health, blood draw fear, and recent nervousness. Analyses of donation frequency and VVR history additionally adjusted for numbers of donations in the three years prior to the exposure assessment period and the previous five years prior to study enrolment respectively.

**Table S4**. Mediation and interaction analyses using differing pain and anxiety severities**.**

| **Analyses of donation experience** | | | | | | |
| --- | --- | --- | --- | --- | --- | --- |
|  | Adjusted^a^ RRs (95% CIs) | | | % mediated | Additive interaction | Multiplicative interaction |
| Mediator |  |  |  |  |  |  |
| Venipuncture pain | TE | NDE | NIE |  |  |  |
| Moderate pain^b^ |  |  |  |  | -0.05 (-0.19, 0.09)  *P* = .51 | 0.92 (0.82, 1.03)  *P* = .17 |
| Severe pain^b^ |  |  |  |  | -0.29 (-0.58, -0.01)  *P* = .04 | 0.77 (0.60, 1.01)  *P* = .06 |
| Donation anxiety |  |  |  |  |  |  |
| Moderate anxiety^c^ | 1.26  (1.20, 1.33) | 1.20  (1.14, 1.26) | 1.04  (1.03, 1.05) | 17.5 (11.5, 23.6) | -0.07 (-0.19, 0.05)  *P* = .26 | 0.89 (0.81, 0.97)  *P* = .01 |
| Severe anxiety^c^ | 1.22  (1.14, 1.30) | 1.22  (1.14, 1.29) | 1.00  (1.00, 1.00) | 0.8 (0.2, 1.4) | 0.31 (-0.19, 0.82)  *P* = .23 | 1.09 (0.80, 1.48) *P* = .59 |
| **Analyses of donation frequency** | | | | | | |
|  | Adjusted^a^ RRs (95% CIs) | | | % mediated | Additive interaction | Multiplicative interaction |
| Mediator |  |  |  |  |  |  |
| Venipuncture pain | TE | NDE | NIE |  |  |  |
| Moderate pain^b^ |  |  |  |  | 0.10 (-0.01, 0.20)  *P* = .07 | 1.04 (0.91, 1.18)  *P* = .58 |
| Severe pain^b^ |  |  |  |  | 0.30 (0.04, 0.56)  *P* = .02 | 1.19 (0.89, 1.58) *P* = .25 |
| Donation anxiety |  |  |  |  |  |  |
| Moderate anxiety^c^ | 1.20  (1.13, 1.26) | 1.17  (1.11, 1.24) | 1.02  (1.01, 1.02) | 10.8 (6.1, 15.5) | 0.02 (-0.08, 0.12)  *P* = .69 | 0.95 (0.86, 1.06)  *P* = .37 |
| Severe anxiety^c^ | 1.17  (1.10, 1.25) | 1.17  (1.10, 1.25) | 1.00  (1.00, 1.00) | 0.1 (-0.1, 0.3) | -0.02 (-0.42, 0.37)  *P* = .91 | 0.93 (0.59, 1.46)  *P* = .76 |
| **Analyses of VVR history** | | | | | | |
|  | Adjusted^a^ RRs (95% CIs) | | | % mediated | Additive interaction | Multiplicative interaction |
| Mediator |  |  |  |  |  |  |
| Venipuncture pain | TE | NDE | NIE |  |  |  |
| Moderate pain^b^ | 1.88  (1.75, 2.00) | 1.87  (1.75, 2.00) | 1.00  (1.00, 1.00) | -0.0 (-0.3, 0.2) | -0.32 (-0.48, -0.15)  *P* < .001 | 0.76  (0.65, 0.89)  *P* < .001 |
| Severe pain^b^ | 1.91  (1.78, 2.05) | 1.91  (1.77, 2.05) | 1.00  (1.00, 1.00) | 0.1 (-0.4, 0.5) | 0.80 (0.59, 1.09)  *P* = .16 | -0.22  (-0.48, 0.04)  *P* = .10 |
| Donation anxiety | TE | NDE | NIE |  |  |  |
| Moderate anxiety^c^ | 2.02  (1.88, 2.18) | 1.90  (1.77, 2.05) | 1.01  (0.99, 1.02) | 1.7 (-0.15, 5.0) | 0.70 (0.61, 0.79)  *P* < .001 | -0.39  (-0.58, -0.19)  *P* = .001 |
| Severe anxiety^c^ | 2.06  (1.87, 2.26) | 2.05  (1.86, 2.26) | 1.00  (1.00, 1.00) | -0.1 (-0.2, 0.1) | 0.51 (0.35, 0.74)  *P* < .001 | -0.98  (-1.32, -0.65)  *P* < .001 |

RR: risk ratio; CI: confidence interval; VVR: vasovagal reaction; TE: total effect, or the association between each donation history exposure and VVR symptom reports; NDE: natural direct effect, or the association between each donation history exposure and VVR symptom reports operating independently of pain or anxiety; NIE: natural indirect effect, or the association between each donation history exposure and VVR symptom reports operating via pain or anxiety.

^a^All analyses adjusted for sex, age, ethnicity, estimated blood volume, diagnosed hypotension, smoking, alcohol intake, leisure time physical activity, general health, blood draw fear, and recent nervousness. Analyses of donation frequency and VVR history additionally adjusted for numbers of donations in the three years prior to the exposure assessment period and the previous five years prior to study enrolment respectively.

^b^Compared with less-than-mild reported pain.

^c^Compared with no reported anxiety.

**Table S5**. Mediation and interaction analyses for donation anxiety using six-item BDAS.

| **Mediator: donation anxiety** | | | | | | |
| --- | --- | --- | --- | --- | --- | --- |
|  | Adjusted^a^ RRs (95% CIs) | | | % mediated | Additive interaction | Multiplicative interaction |
| Exposure | TE | NDE | NIE |  |  |  |
| Donation experience | 1.26  (1.20, 1.32) | 1.22  (1.16, 1.27) | 1.03  (1.02, 1.04) | 13.7 (9.6, 17.9) | -0.09 (-0.45, 0.27)  *P* = .62 | 0.71 (0.59, 0.86)  *P* < .001 |
| Donation frequency | 1.19  (1.13, 1.26) | 1.16  (1.10, 1.23) | 1.02  (1.02, 1.03) | 14.3 (8.80, 19.8) | 0.12 (0.04, 0.21)  *P* = .005 | 0.98 (0.87, 1.11)  *P* = .76 |
| VVR history | 1.90  (1.78, 2.03) | 1.81  (1.69, 1.93) | 1.02  (1.01, 1.04) | 5.1 (1.7, 8.5) | -0.09 (-0.45, 0.27)  *P* = .62 | 0.71 (0.59, 0.86) *P* < .001 |

RR: risk ratio; CI: confidence interval; VVR: vasovagal reaction; TE: total effect, or the association between each donation history exposure and VVR symptom reports; NDE: natural direct effect, or the association between each donation history exposure and VVR symptom reports operating independently of pain or anxiety; NIE: natural indirect effect, or the association between each donation history exposure and VVR symptom reports operating via pain or anxiety.

^a^All analyses adjusted for sex, age, ethnicity, estimated blood volume, diagnosed hypotension, smoking, alcohol intake, leisure time physical activity, general health, blood draw fear, and recent nervousness. Analyses of donation frequency and VVR history additionally adjusted for numbers of donations in the three years prior to the exposure assessment period and the previous five years prior to study enrolment respectively.

**Table S6**. Mediation and interaction analyses using a complete-case approach.

| **Mediator: venipuncture pain** | | | | | | |
| --- | --- | --- | --- | --- | --- | --- |
|  | Adjusted^c^ RRs (95% CIs) | | | % mediated | Additive interaction | Multiplicative interaction |
| Exposure | TE | NDE | NIE |  |  |  |
| Donation experience^a^ | – | – | – | – | -0.08 (-0.18, 0.02)  *P* = 0.10 | 0.90 (0.80, 1.01)  *P* = 0.08 |
| Donation frequency^b^ | – | – | – | – | 0.13 (-0.02, 0.28)  *P* = 0.10 | 1.05 (0.92, 1.20)  *P* = 0.43 |
| VVR history | 1.84 (1.72, 1.98) | 1.84 (1.71, 1.97) | 1.00 (1.00, 1.00) | 0.0 (-0.5, 0.6) | -0.28 (-0.43, -0.13)  *P* < 0.001 | 0.78 (0.66, 0.91)  *P* = 0.002 |
| **Mediator: donation anxiety** | | | | | | |
|  | Adjusted^c^ RRs (95% CIs) | | | % mediated | Additive interaction | Multiplicative interaction |
| Exposure | TE | NDE | NIE |  |  |  |
| Donation experience | 1.28 (1.21, 1.35) | 1.21 (1.15, 1.28) | 1.04 (1.03, 1.05) | 18.2 (11.8, 24.5) | -0.03 (-0.14, 0.07)  *P* = 0.52 | 0.91 (0.82, 1.00)  *P* = 0.05 |
| Donation frequency | 1.21 (1.14, 1.28) | 1.18 (1.12, 1.26) | 1.02 (1.01, 1.02) | 10.3 (5.6, 15.0) | -0.01 (-0.16, 0.15)  *P* = 0.94 | 0.93 (0.83, 1.04)  *P* = 0.23 |
| VVR history | 1.97 (1.82, 2.13) | 1.84 (1.70, 1.99) | 1.01 (1.00, 1.04) | 2.9 (-1.1, 6.9) | -0.33 (-0.52, -0.15)  *P* < 0.001 | 0.71 (0.62, 0.82)  *P* < 0.001 |

RR: risk ratio; CI: confidence interval; TE: total effect, or the association between each donation history exposure and VVR symptom reports; NDE: natural direct effect, or the association between each donation history exposure and VVR symptom reports operating independently of pain or anxiety; NIE: natural indirect effect, or the association between each donation history exposure and VVR symptom reports operating via pain or anxiety.

^a^Mediation analyses not conducted because donation experience showed a weak inverse association with venipuncture pain following covariate adjustment.

^b^Mediation analyses not conducted because donation frequency showed a null association with venipuncture pain following covariate adjustment.

^c^All analyses adjusted for sex, age, ethnicity, estimated blood volume, diagnosed hypotension, smoking, alcohol intake, leisure time physical activity, general health, blood draw fear, and recent nervousness. Analyses of donation frequency and VVR history additionally adjusted for numbers of donations in the three years prior to the exposure assessment period and the previous five years prior to study enrolment respectively.

**References**

1 van Buuren, S., Boshuizen, H. C. & Knook, D. L. Multiple imputation of missing blood pressure covariates in survival analysis. *Statistics in Medicine* **18**, 681-694 (1999). <https://doi.org/https://doi.org/10.1002/(SICI)1097-0258(19990330)18:6><681::AID-SIM71>3.0.CO;2-R

2 White, I. R., Royston, P. & Wood, A. M. Multiple imputation using chained equations: Issues and guidance for practice. *Stat Med* **30**, 377-399 (2011). <https://doi.org/10.1002/sim.4067>

3 van Buuren, S. & Groothuis-Oudshoorn, K. mice: Multivariate Imputation by Chained Equations in R. *Journal of Statistical Software* **45**, 1 - 67 (2011). <https://doi.org/10.18637/jss.v045.i03>

4 Collins, L. M., Schafer, J. L. & Kam, C. M. A comparison of inclusive and restrictive strategies in modern missing data procedures. *Psychol Methods* **6**, 330-351 (2001).

5 Tilling, K., Williamson, E. J., Spratt, M., Sterne, J. A. & Carpenter, J. R. Appropriate inclusion of interactions was needed to avoid bias in multiple imputation. *J Clin Epidemiol* **80**, 107-115 (2016). <https://doi.org/10.1016/j.jclinepi.2016.07.004>

6 Heymans, M. W. & Twisk, J. W. R. Handling missing data in clinical research. *Journal of Clinical Epidemiology* **151**, 185-188 (2022). <https://doi.org/https://doi.org/10.1016/j.jclinepi.2022.08.016>

7 Buuren, S. *Flexible Imputation of Missing Data, Second Edition*. (2018).

8 Morris, T. P., White, I. R. & Royston, P. Tuning multiple imputation by predictive mean matching and local residual draws. *BMC Medical Research Methodology* **14**, 75 (2014). <https://doi.org/10.1186/1471-2288-14-75>

9 Moore, R. A., Straube, S. & Aldington, D. Pain measures and cut-offs – ‘no worse than mild pain’ as a simple, universal outcome. *Anaesthesia* **68**, 400-412 (2013). <https://doi.org/https://doi.org/10.1111/anae.12148>

10 Zsido, A. N., Teleki, S. A., Csokasi, K., Rozsa, S. & Bandi, S. A. Development of the short version of the spielberger state—trait anxiety inventory. *Psychiatry Research* **291**, 113223 (2020). <https://doi.org/https://doi.org/10.1016/j.psychres.2020.113223>

11 Chell, K., Waller, D. & Masser, B. The Blood Donor Anxiety Scale: a six-item state anxiety measure based on the Spielberger State-Trait Anxiety Inventory. *Transfusion* **56**, 1645-1653 (2016). <https://doi.org/10.1111/trf.13520>
